# Evaluation of Bai-Zhu (*Atractylodes macrocephala*)-based herbal formulae in breast cancer: Implications for metastasis via Zeb1 and Slug modulation

**DOI:** 10.37796/2211-8039.1704

**Published:** 2026-06-01

**Authors:** Yen Shen Lu, Pei Yen Yeh, Ling-Chun Yeh, Chia-Lin Lee, Ying-Chyi Song

**Affiliations:** a Department of Oncology, National Taiwan University Hospital, Taipei, Taiwan; b TCM Division, Jin-Mi Company, Taipei, Taiwan; c Department of Cosmeceutics, China Medical University, Taichung, Taiwan; d Chinese Medicine Research and Development Center, China Medical University Hospital, Taichung, Taiwan; e Chinese Medicine Research Center, China Medical University, Taichung, Taiwan; f Graduate Institute of Integrated Medicine, College of Chinese Medicine, China Medical University, Taichung, Taiwan

**Keywords:** *Atractylodes macrocephala* Koidz. [Asteraceae], *Cynanchi Atrati Radix*, Jia Wei Xiao Yao San, Breast cancer, Metastasis

## Abstract

**Background:**

Bai-Zhu (*Atractylodes macrocephala* Koidz. [Asteraceae]) is classified in traditional Chinese medicine (TCM) as an herb that targets the gastric and pancreatic meridians to support normal nutrient uptake. As such, Bai-Zhu is commonly included in herbal formulations for treating gastrointestinal dysfunction and alleviating pain and swelling. Formulae containing Bai-Zhu are also frequently used as complementary or alternative therapies to mitigate the side effects of tumor treatments. However, their direct effects on cancer cells remain inadequately understood in modern biomedical science. This study aims to characterize the impact of Bai-Zhu-containing formulae on breast cancer cells using contemporary molecular and cellular biology approaches.

**Methods:**

A tetrazolium-based semiautomated colorimetric (MTT) assay was used to assess cell viability. Western blotting was performed to evaluate protein expression. Quantitative RT-PCR was employed to measure the relative expression levels of selected genes. A Matrigel invasion assay was used to assess the invasive capacity of cancer cells.

**Results:**

Water extracts of Bai-Zhu or formulae containing Bai-Zhu increased the expression of metastasis-related proteins Zeb1 and Slug in breast cancer cells. Moreover, these formulae further enhanced Zeb1 and Slug expression when combined with the chemotherapeutic drug doxorubicin. Matrigel invasion assays confirmed that these treatments promoted the invasive activity of breast cancer cells.

**Conclusions:**

The use of Bai-Zhu-containing formulae should be carefully evaluated, particularly in cancer patients who use them as complementary or alternative treatments, due to their potential to promote tumor metastasis.

## 1. Introduction

According to traditional Chinese medicine, Bai-Zhu (*Atractylodes macrocephala* Koidz. [Asteraceae], BZ) affects the spleen and stomach meridians, which indicates that BZ is involved in the function of the digestive system or in nutrient uptake. Due to its biological effects, BZ is commonly used in combination with other medicinal herbs to form a multicomponent formula that is used to treat various pathophysiological conditions, including loss of appetite, abdominal distension, and diarrhea. Additionally, some BZ-containing formulae have been employed in tumor therapy to mitigate the side effects of therapeutic drugs. It has also been reported to have anti-inflammatory and anticancer activities [1–3].

In Taiwan, approximately 300–500 metric tons of BZ are imported from China each year (data obtained from https://portal.sw.nat.gov.tw_〉_ APGA). According to records from the Ministry of Health and Welfare, among the top 30 most frequently used Chinese medical formulae, six herbal formulae containing BZ are Jia Wei Xiao Yao San (rank 1), Buzhong Yiqi Tang (rank 2), Xiang Sha Liu Jun Zi Tang (rank 9), Guipi Tang (rank 20), Danggui Shaoyao Tang (rank 25), and Huo Xiang Zheng Qi San (rank 30). In addition, Jia Wei Xiao Yao San was shown to be the first choice for breast cancer patients who used Chinese herbal formulae as supplemental and/or alternative treatments [4]. In 2021, twelve thousand metric tons of BZ and several hundred metric tons of BZ-containing herbal formulae were imported from China into the USA (information obtained from https://www.zauba.com/USA-import-data-analysis). The suggested dose of BZ-containing formula is 5 g/day; based on this dose, the imported BZ and BZ-containing formulae are sufficient for a one-year use of 7–8 million people. Members of the general public can easily obtain BZ-containing formulae from community pharmacies or online. Individuals can ingest these formulae without any specific restrictions. However, based on the principle of traditional Chinese medicine known as “one person, one prescription”, those BZ-containing formulae may cause different effects due to the different physiological conditions of each individual. How to use these herbal formulae should be further identified.

According to a principal rule “jun, chen, zuo, and shi” in traditional Chinese medicine, the traditional Chinese herbal formulae are usually composed of multiple herbal components, including principal effective components and components affecting the decrease in side effects or enhancing the speed of drug delivery in the body. Because of the existence of multiple components, a Chinese herbal formula could target various important molecules and processes in different types of tumors [5]. This unique character might be helpful for tumor therapy; however, the disadvantage is that it is hard to characterize the underlying mechanism of its effect on the basis of modern molecular and cellular biology. Identifying the main effective molecule is a way to help traditional Chinese herbal formulae fit into modern medicine.

Numerous chemicals extracted from different Chinese medicinal herbs have been characterized to have benefits for tumor therapy, either alone or combined with modern tumor therapy [5]. For example, curcumin, a polyphenolic chemical extracted from turmeric of Curcuma longa, has been identified to have a broad range of biological effects, including anti-inflammation and antioxidant effects [6]. Curcumin has been shown to inhibit the growth of breast cancer cells in culture and in clinical trials [7,8]. Most chemicals extracted from medicinal herbs might face a similar problem, in that the bioavailability of such chemicals is low, thereby reducing their effectiveness [9]. Much effort has been given to develop a drug delivery system to improve their availability. For example, nano-capsules with different compositions have been demonstrated to increase the biological effects of curcumin [10]. These attempts play an important role in the development of novel strategies for tumor therapy.

The oncogenic protein Zeb1 may play an important role in tumor metastasis by promoting tumor cell motility and it also induces epithelial-mesenchymal transition. Numerous molecules associated with metastasis are affected through Zeb1-mediated positive or negative regulation [5,11]. PD-L1 serves as a “don’t find me” signal that helps tumor cells escape host immune surveillance. It has become an important target in the recent rapidly developing strategy of immunotherapy [12,13]. We previously reported that BZ can increase the expression of estrogen receptor (ER), Slug, and PD-L1 in breast cancer cells [14], suggesting that BZ may influence tumor growth, metastasis, and immune evasion. Because BZ-containing formulae are widely used, the identification of the biological effects of these BZ-containing formulae on the protein levels of Zeb1 and PD-L1 in tumor cells may be important. In this study, we found that BZ-containing formulae increased the protein expression levels of Zeb1 and PD-L1 in breast cancer cells and that combined treatment with the anticancer drug doxorubicin further enhanced this effect. The Matrigel assay demonstrated increased invasive activity of cancer cells following treatment with these formulae alone or in combination with doxorubicin. Our study suggests that the use of BZ-containing formulae should be carefully evaluated on an individual basis, particularly in patients with tumors.

## 2. Materials and methods

### 2.1. Cell culture and chemicals

The culture medium for the cells that were used in this study was DMEM for MCF7 cells and DMEM/F12 for MDA-MB231 cells. The medium was supplemented with 10% fetal bovine serum (FBS). The cells were incubated in a humidified incubator with 5% CO_2_ at 37 °C.

### 2.2. Preparation of the herb extract

The BZ-containing formulae (Si Jun Zi Tang, Jia Wei Xiao Yao San, Shen Ling Bai Zhu San, and Huo Xiang Zheng Qi San) were purchased from Sun Ten Pharmaceutical Co. (Taiwan). These herbal formulae are classic Chinese herbal formulae and recorded in Chinese medical books. The composition of each formula is listed in Supplementary File 1 (https://www.biomedicinej.com/cgi/editor.cgi?window=additional_files&article=1704&context=biomedicine). For reconstitution of the formula, each component was prepared from dried material and obtained from a commercial Chinese medical herbal store. The quality and identity of this herb are restricted by the Food and Drug Administration of Taiwan. Voucher specimens for each herb were retained. The four BZ-containing formulae were dissolved in water such that the concentration of BZ was 25 mg/mL. A single dried herb was prepared at 100 mg/mL in water. All of the preparations were autoclaved, and the solution was stored at −20 °C. The indicated concentration of each preparation represented the concentration of BZ in each formula, and the concentrations of the other components were determined according to the defined relative ratio to BZ. The identities of the BZ and BW water extracts were determined via high-performance liquid chromatography (HPLC) (the detailed methods and results are shown in Supplementary Figures S1 and S2 (https://www.biomedicinej.com/cgi/editor.cgi?window=additional_files&article=1704&context=biomedicine)) in the laboratory.

### 2.3. Evaluation of cell number

A tetrazolium-based semiautomated colorimetric assay (MTT assay) was used to determine the number of surviving cells. The cells were subjected to various treatments for 3 days, as indicated in the figures. The number of cells was evaluated via the MTT assay with an ELISA reader at OD_540_.

### 2.4. Western blot analysis

Western blot analysis was used to determine the protein expression. After various treatments (as indicated in the figures), the cells were lysed with RIPA buffer containing a cocktail of protease inhibitors. Aliquots of lysates were subjected to Western blotting analysis. All of the antibodies were purchased from Cell Signaling Technology. The images were developed with a chemiluminescence reagent.

### 2.5. Quantitative RT-PCR

RNA was extracted via an RNeasy Mini Kit (Qiagen), and cDNA was synthesized via reverse transcription. The relative expression level of the gene was determined via SYBR Green PCR Master Mix on an Azure Cileo real-time PCR machine (Azure Biosystems). The utilized primers were GAPDH: forward, 93TCGGAGTCAACGGATTTGGT and reverse, 273TTCCCGTTCTCACCCTTGAC (Sequence ID: NM_002046.7); Zeb1: forward, 789CCTGTCCATATTGTGATAGAGGC and reverse, 983ACCCAGACTGCGTCACATGT (Sequence ID: NM_001128128.3); Slug: forward, 615TGCGATGCCCAGTCTAGAAA and reverse, 796TTCTCCCCCGTGTGAGTTC (Sequence ID: NM_003068.5); and PD-L1: forward, 51TGCAGGGCATTCCAGAAAGA and reverse, 137ACCGTGACAGTAAATGCGTTC (Sequence ID: NM_014143.4). The PCR reaction parameters were 95 °C for 10 min, followed by 40 cycles of 95 °C for 15 s and 60 °C for 1 min. The relative expression level of the target gene was calculated via the ΔCt (threshold cycle) method: relative expression = 2-ΔCt, where ΔCt = Ct (target gene) - Ct (control gene). The GAPDH gene was used as an input control. The specificity of the primer set for the target gene was demonstrated via analysis of the dissociation curve (data not shown).

### 2.6. Matrigel analysis

The invasive ability of cancer cells was evaluated according to an internet-available transwell assay protocol (https://pharm.ucsf.edu_〉_ xinchen _〉_media browser). Briefly, the cancer cells were seeded into a Matrigel precoated 8-mm pore size transwell plate and then treated with various drugs (as indicated in the figure legends) for 24 h. The cells were fixed with methanol and stained with Giemsa solution. The cells on the upper side of the membrane were removed, and the cells on the lower side of the membrane were counted under a microscope at 40× magnification.

## 3. Results

### 3.1. BZ and BZ-containing formulae increase the protein levels of Zeb1 and Slug in MCF7 cells

**Fig. 1:**
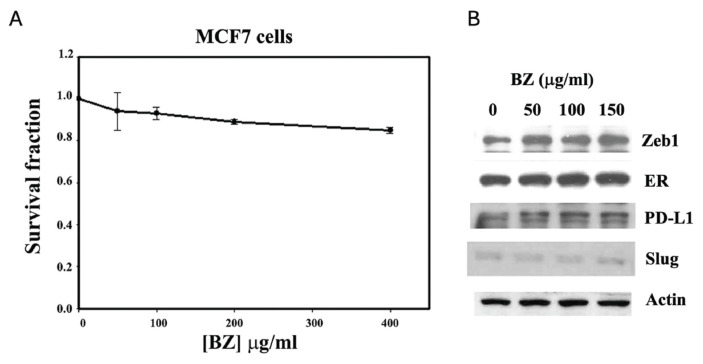
(A). The effect of the herb BZ on the growth of MCF7 cells. The MTT assay was used to determine the number of surviving cells after a serial dose treatment of BZ. “Data are presented as the survival fraction relative to untreated controls (mean ± SD). (B) Effects of the herb BZ on the protein expression of MCF7 cells. MCF7 cells were treated with various doses of BZ for 24 h. The lysates were prepared, and Western blot analysis was used to characterize the protein expression levels. The actin stain served as a loading control.

We characterized the potential effect of BZ on the breast cancer cell line MCF7. We found that BZ had only a slight effect on the growth of MCF7 cells (Fig. 1A). However, BZ increased the ER and PD-L1 protein levels in a dose-dependent manner (Fig. 1B). We further characterized the effect of BZ on the expression of the metastasis-related proteins Zeb1 and Slug. BZ increased the level of Zeb1 but had no effect on Slug expression (Fig. 1B).

**Fig. 2:**
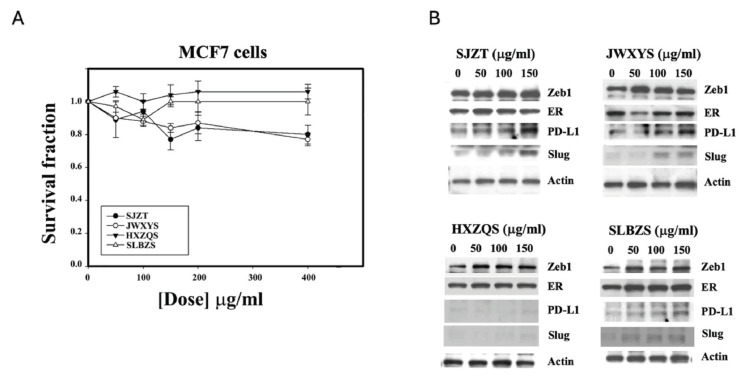
(A) MCF7 cells were treated with four BZ-containing formulae for 3 days, after which an MTT assay was used to evaluate the number of surviving cells. The data are shown as the survival fraction of the untreated control and are presented as the means ± SD. (B) MCF7 cells were treated with various doses of BZ-containing formulae for 24 h. Western blot analysis was used to characterize the levels of protein expression.

Because BZ-containing formulae are often used by tumor patients, we selected four BZ-containing formulae, including Jia Wei Xiao Yao San (JWXYS), Si Jun Zi Tang (SJZT), Shen Ling Bai Zhu San (SLBZS), and Huo Xiang Zheng Qi San (HXZQS), and characterized the effects of these formulae on breast cancer cell lines. As shown in Fig. 2A, the four herbal formulae exhibited little to no effect on the growth of MCF7 cells. However, they significantly influenced the expression of several proteins, including Zeb1, ER, PD-L1, and Slug, in MCF7 cells to varying degrees (Fig. 2B). Specifically, SJZT markedly increased the expression of PD-L1 and Slug, JWXYS elevated the expression of Zeb1, PD-L1, and Slug, HXZQS enhanced the expression of Zeb1, and SLBZS increased the expression of Zeb1, ER, PD-L1, and Slug.

**Fig. 3:**
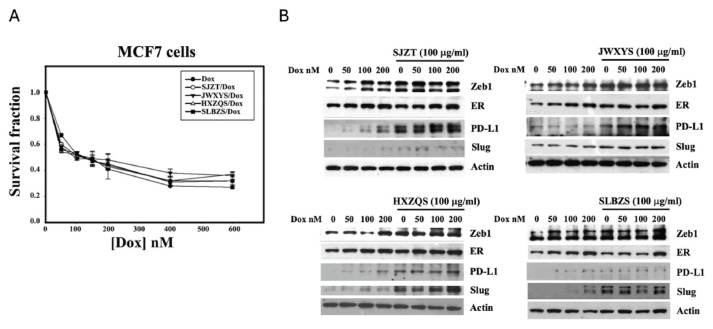
(A) MCF7 cells were treated with various dose of doxorubicin alone or with 4 different BZ-containing formulae (100 μg/mL) for 3 days, after which an MTT assay was used to evaluate the number of surviving cells. The data are shown as the survival fraction of the untreated control and are presented as the means ± SD. (B) MCF7 cells were treated with serial doses of doxorubicin either alone or with 4 different BZ-containing formulae (100 μg/mL) for 24 h. Western blot analysis was used to characterize the levels of protein expression.

We further characterized the combined effect of these BZ-containing formulae with doxorubicin on MCF7 cells. The four BZ-containing herbal formulae did not affect the cytotoxic effect of doxorubicin on MCF7 cells (Fig. 3A). However, when in combination with doxorubicin treatment, these formulae further enhanced the expression of Zeb1, PD-L1, and Slug proteins compared to doxorubicin treatment alone, with the exceptions that SJZT did not markedly influence Slug expression and SLBZS had no effect on PD-L1 expression (Fig. 3B).

### 3.2. The effects of BZ-containing formulae on the MDAMB231 cells

**Fig. 4:**
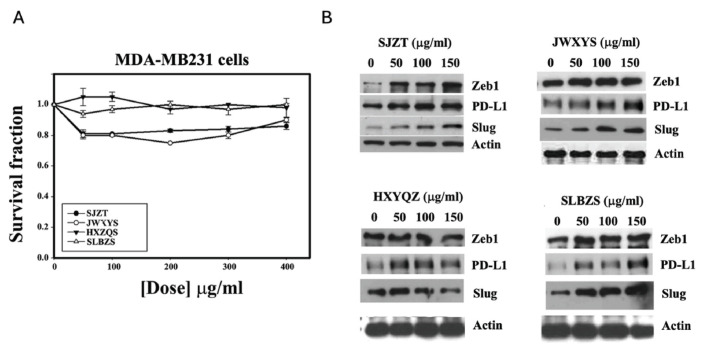
The effects of four different BZ-containing formulae on the growth (A) and protein expression (B) of MDA-MB231 cells. The treatment of the MDA-MB231 cells was the same as that of the MCF7 cells.

The four herbal formulae were further used to treat the triple-negative breast cancer cell line MDA-MB231. Consistent with the findings in MCF7 cells, the four BZ-containing formulae exhibited a slight effect on the growth of MDA-MB231 cells (Fig. 4A). Similarly, these formulae significantly altered the expression of proteins, including Zeb1, PD-L1, and Slug, in MDA-MB231 cells to varying degrees (Fig. 4B). Specifically, SJZT markedly increased the expression of Zeb1, PD-L1, and Slug; JWXYS elevated the expression of Zeb1, PD-L1, and Slug; HXZQS enhanced PD-L1 expression; and SLBZS increased the levels of Zeb1, PD-L1, and Slug.

### 3.3. BZ-containing formulae increase the drug resistance of MDA-MB231 cells to doxorubicin

**Fig. 5:**
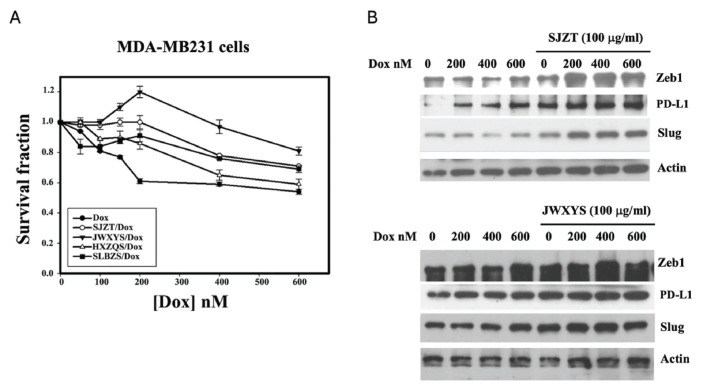
MDA-MB231 cells were treated as indicated. The MTT assay was used to evaluate the number of surviving cells (A), and Western blot analysis was used to characterize the levels of protein expression (B).

The effects of the BZ-containing formulae in combination with doxorubicin on MDA-MB231 cells were also determined. Fig. 5A shows that these four herbal formulae increased the resistance of MDA-MB231 cells to doxorubicin. The formulae SJZT and JWXYS (which are widely used by breast cancer patients) were selected for further characterization. As shown in Fig. 5B, both formulae (especially SJZT) enhanced the upregulating effect of doxorubicin on the expression of Zeb1, PD-L1, and Slug in MDA-MB231 cells.

### 3.4. Searching for herbs that can replace BZ

**Fig. 6:**
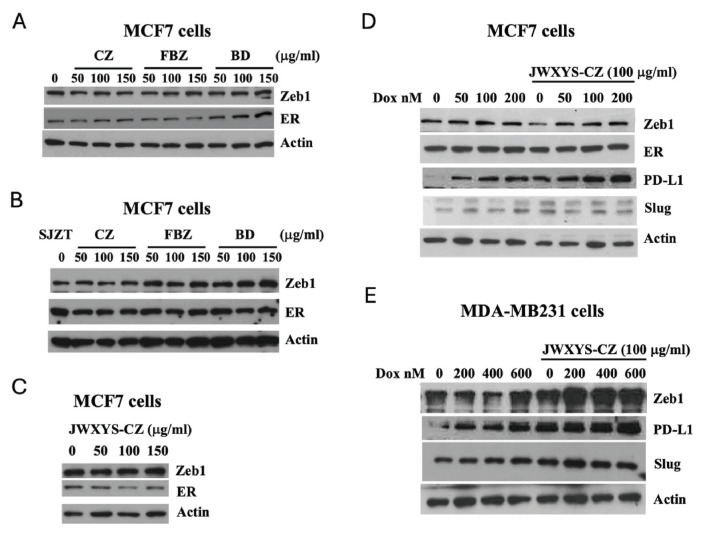
MCF7 cells were treated with a serial dose of (A) three different herbs (CZ, FBZ, and BD) or (B) SJZT, in which BZ was replaced with CZ, FBZ, and BD for 24 h. (C) MCF7 cells were treated with serial doses of JWXYS-CZ for 24 h. (D) MCF7 cells were treated with a serial dose of either doxorubicin alone or with 100 μg/mL JWXYS-CZ for 24 h. (E) MDA-MB231 cells were treated with serial doses of doxorubicin alone or with 100 μg/mL JWXYS-CZ for 24 h. Western blot analysis was used to characterize the levels of protein expression.

We attempted to find other herbs that could replace the BZ of the investigated formulae. Three medicinal herbs, including Chang Zhu (Rhizoma Atractylodis; CZ), fried BZ (FBZ), and Bian Dou (Dolichos lablab L.; BD), which have similar characteristics to BZ in Chinese medicine, were used either alone or in place of BZ in the SJZT formula to treat MCF7 cells. Western blotting analysis revealed that either CZ alone or CZ incorporated into the SJZT formula did not increase the protein levels of Zeb1 or ER (Fig. 6A and B). We also reconstituted JWXYS by replacing BZ with CZ. The new formula JWXYS-CZ alone had little effect on the protein levels of both Zeb1 and ER in MCF7 cells (Fig. 6C). We further characterized the combined effect of JWXYS-CZ and doxorubicin on both MCF7 and MDA-MB231 cells (Fig. 6D and E). JWXYS-CZ combined with doxorubicin specifically increased PD-L1 protein levels but had no effect on doxorubicin-induced expression of Zeb1, ER, or Slug in MCF7 cells. In contrast, JWXYS-CZ increased doxorubicin-induced Zeb1 and PD-L1 protein expression but did not affect Slug expression in MDA-MB231 cells. This result suggests that CZ is not a suitable herb for replacing BZ.

**Fig. 7:**
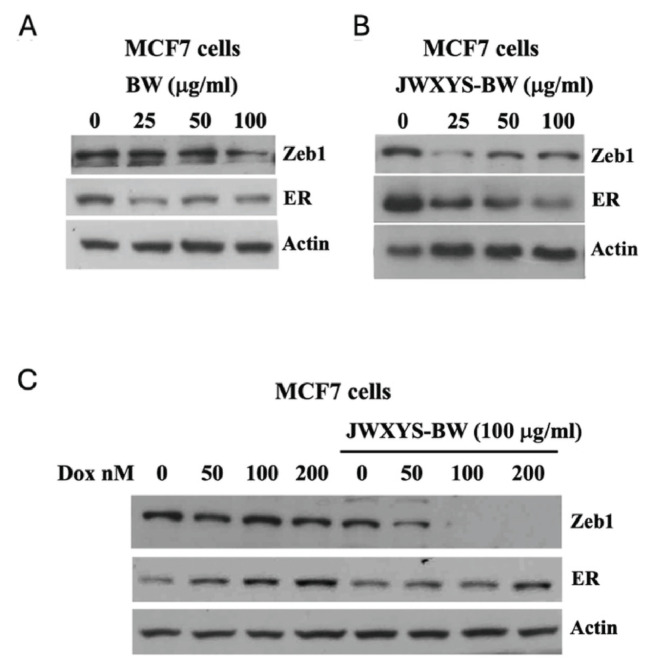
MCF7 cells were treated with a serial dose of (A) BW, (B) JWXYS-BW for 24 h or (C) doxorubicin alone or with 100 μg/mL JWXYS-BW for 24 h. Western blot analysis was used to characterize the levels of protein expression.

We previously reported that the herb Bai-Wei (*Radix Cynanchi atrati*; BW) decreases the growth of various cancer cells and the expression of several proteins, including ER, PD-L1, and Slug [14]. We used the herb BW to replace BZ in the formula JWXYS. Western blot analysis revealed that Zeb1 and ER expression in MCF7 cells was suppressed by BW alone or by the JWXYS-BW treatment (Fig. 7A and B). Importantly, JWXYS-BW also decreased the doxorubicin-induced proteins Zeb1 and ER expression (Fig. 7C). The protein levels of PD-L1 and Slug were undetectable under these treatments, which was likely due to the low basal expression of both proteins in untreated MCF7 cells (data not shown).

**Fig. 8:**
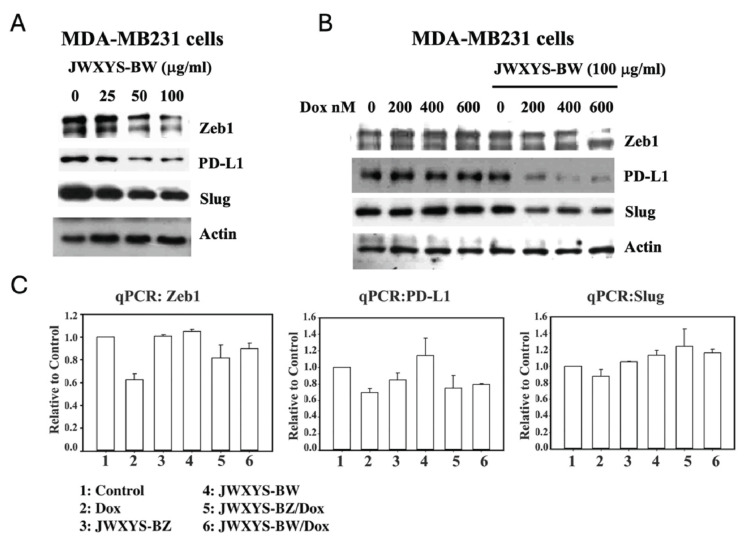
MDA-MB231 cells were treated with (A) a serial dose of JWXYS-BW or (B) a serial dose of doxorubicin alone or with 100 μg/mL JWXYS-BW for 24 h. Western blot analysis was used to characterize the levels of protein expression. (C) MDA-MB231 cells were treated with 1. Control, 2. 400 nM doxorubicin, 3. 100 μg/mL JWXYS-BZ, 4. 100 μg/mL JWXYS-BW, 5. 400 nM doxorubicin and 100 μg/mL JWXYS-BZ, and 6. 400 nM doxorubicin and 100 μg/mL JWXYS-BW for 24 h qPCR was performed to determine the relative RNA level of each gene. The standard deviations are shown in the figure.

The formula JWXYS-BW also decreased the basal and doxorubicin-induced protein levels of Zeb1, PD-L1 and Slug in MDA-MB231 cells (Fig. 8A and B). To identify the cause of the changes in the expression of these proteins, qPCR was performed to determine the relative RNA level of each protein in the cells subjected to different treatments, as shown in Fig. 8C. All of the treatments did not significantly affect the RNA level of each targeted gene when the cutoff value was set at 2-fold (which represents a general cutoff value). Both JWXYS-BZ and JWXYS-BW slightly reversed the doxorubicin-induced decrease in the RNA levels of Zeb1 and Slug. These results suggest that the change of protein level under these treatments was not regulated at the transcriptional level and that other mechanisms may be involved.

### 3.5. JWXYS-BW suppresses MDA-MB231 cell invasion

**Fig. 9:**
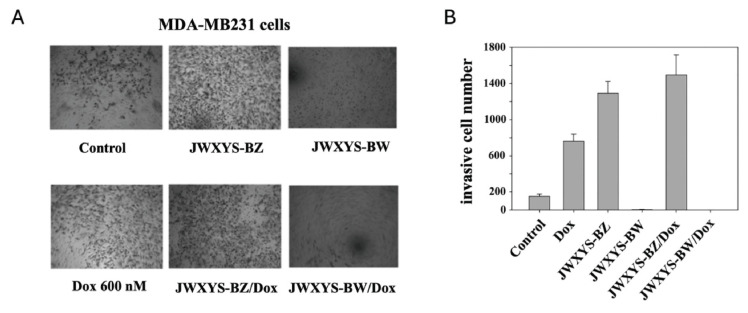
Determination of the invasive activity of MDA-MB231 cells via Matrigel analysis. MDA-MB231 cells were treated as in the qPCR experiments for 24 h. The stained cells on the lower side of the membrane were photographed under a microscope at 40 × magnification (A), and (B) the number of cells was counted. The data were obtained from two independent experiments and are presented as the means ± SD.

The Matrigel assay was employed to determine the invasive activity of MDA-MB231 cells under different treatments. As shown in Fig. 9, doxorubicin increased the invasive activity of MDA-MB231 cells. JWXYS-BZ alone increased invasive activity. Moreover, the combination of JWXYS-BZ and doxorubicin further enhanced the invasive activity of the MDA-MB231 cells. In contrast, JWXYS-BW significantly suppressed not only the basal invasive activity but also the doxorubicin-induced invasive activity of MDA-MB231 cells.

### 3.6. High-dose BZ-containing formula inhibits cell growth and related protein expression

**Fig. 10:**
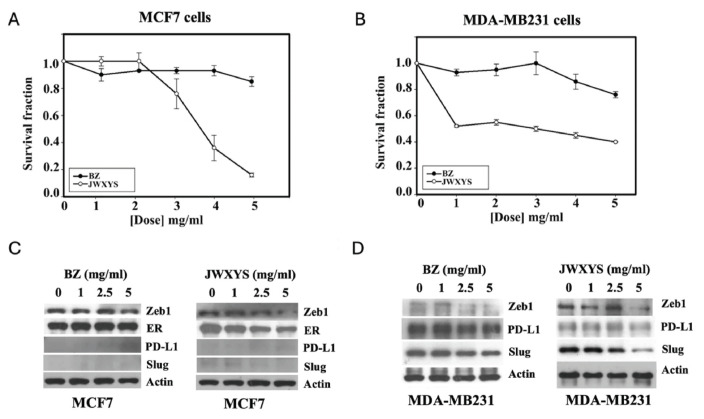
High-dose effects of BZ and the formula JWXYS. (A) MCF7 and (B) MDA-MB231 cells were treated with a serial dose of BZ or JWXYS for three days. The number of surviving cells was evaluated via the MTT assay. The data are shown as the survival fraction of the untreated control and are presented as the means ± SD. (C) MCF7 and (D) MDA-MB231 cells were treated with a serial dose of BZ or JWXYS for 24 h. Western blot analysis was used to characterize the levels of protein expression.

Our results differ from previous reports, in which BZ was shown to have anticancer activity [1,15,16]. On the other hand, Chen et al. reported that JWXYS had no cytotoxic effect on MCF7 cells [17]. To address these conflicting results, we applied a higher dose range (at the mg/mL level) of BZ or the formula JWXYS to treat MCF7 and MDA-MB231 breast cancer cells. As shown in Fig. 10, both BZ and the formula JWXYS reduced cell growth and target protein expression in both types of breast cancer cells.

## 4. Discussion

A rapidly increasing number of studies have shown that the Chinese herbal formula can affect various cellular processes, which is likely achieved via multiple components of the herbal formula [18,19]. Interestingly, a single chemical extracted from a medical herb is shown to target multiple molecules and affect various processes in different types of tumors. For example, curcumin decreases the expression level of Myc, Ras, EGFR, and suppresses angiogenesis, cancer-associated fibroblasts, and inflammation-related processes in colon, breast, and prostate cancers. Many other chemicals, such as berberine, resveratrol, and baicalin, have also been identified to exert multi-targeting activity [5,20]. The underlying mechanism remains to be further characterized. However, cellular physiological conditions are regulated by a complex molecular network. The intercellular or intracellular stimuli may induce numerous promotive and inhibitory processes, and/or influence the equilibrium among those processes. The outcomes are determined by the sum of all the altered biochemical processes. This scenario increases the difficulty of identifying the underlying mechanisms of a specific herbal formula. Furthermore, various new physical and chemical risk factors cause human diseases in different ways; thus, it is difficult to use a single formula to treat various abnormal physiological conditions of the human body. The re-evaluation of traditional herbal formulae usage on a modern biochemical and molecular basis may be necessary for specific physiological conditions. This study aligns with the scope of the Special Issue “4Rs in Ethnopharmacology,” as it contributes to refining and reassessing traditional herbal formulae to enhance their safety and efficacy. Our research exclusively utilizes cell lines as experimental models, allowing controlled investigation of the mechanistic effects of these herbal formulae. We have sufficient cellular molecular data to support the conclusions drawn in this study, providing robust evidence for the impact of BZ and its alternative formulation in cancer therapy.

In this study, we showed that the BZ-containing formulae increased ER and PD-L1 expression. We further demonstrated that the BZ-containing formula increased Zeb1 and Slug protein levels, along with the subsequent invasive activity in breast cancer cells. This effect was further enhanced when BZ was combined with the anticancer drug doxorubicin. Notably, BZ-containing formulae (particularly JWXYS) are often administered to tumor patients.

We showed that a high concentration (mg/mL) of JWXYS inhibited the growth and expression of target proteins in breast cancer cells. The most likely explanation for this result may be that uncertain inhibitory factors in the JWXYS extract were present in low amounts and/or required high threshold concentrations to activate their downstream processes. In contrast, the factor promoting protein expression was at a high amount or had a low threshold concentration to initiate the downstream reaction. The inhibitory components subsequently became dominant determinants once their amount reached a high enough concentration to activate their downstream processes.

However, the distribution of anticancer drugs is not homogenous, which is due to interstitial fluid pressure in the tumor mass [21–24]. Some tumor cells (particularly in large tumors) are exposed to low-dose or sublethal-dose anticancer drug treatments. A similar scenario likely applies to herbal formulae. Given that a cancer patient with a large tumor may have a greater inclination to use complementary or alternative treatments, our study suggests that the BZ-containing formula may not be suitable for tumor patients.

In this study, we showed that the herb BW-containing formulae (both SJZT-BW and JWXYS-BW) inhibited the protein expression of several proteins, including ER, PD-L1, Zeb1, and Slug, which play critical roles in tumor progression, metastasis, and the tumor response to host immunosuppression. Based on these findings, both SJZT-BW and JWXYS-BW are likely more suitable for cancer patients who are considering herbal formulae as part of their treatment. However, the BZ-containing formula has been used to treat various pathological conditions, particularly related to the digestive system. Some BZ-containing formulae have specific uses; for example, SJZT is used to treat functional (nonulcer) dyspepsia [25]. Furthermore, the JWXYS formula functions in treating some neurological disorders, emotional depression, and premenstrual syndrome [26–28]. Whether SJZT-BW and JWXYS-BW retain the original functions remains uncertain and warrants clinical validation.

## 5. Conclusions

BZ-containing formulae, such as JWXYS, are widely used throughout the world. However, based on the results of our present study, we suggest that BZ-containing formulae should be used with caution, particularly for tumor patients who are receiving chemotherapy and/or immunotherapy. The TCM principle of ‘one person, one prescription’ aligns with modern ‘personalized medicine’. We do not intend to replace BZ-containing formulae with BW-containing formulae in all aspects of treatment.

## Supplementary Information





## Data Availability

The raw data supporting the conclusions of this article will be made available by the authors without undue reservation.

## List of abbreviations

BZ: Bai-Zhu (*Atractylodes Macrocephala* Koidz)
Zeb1: Zinc Finger E-box Binding Homeobox 1
PD-L1: Programmed Death-Ligand 1
ER: Estrogen Receptor
DMEM: Dulbecco’s Modified Eagle Medium
FBS: Fetal Bovine Serum
MTT assay: Tetrazolium-Based Semiautomated Colorimetric Assay
RIPA buffer: Radio-Immunoprecipitation Assay Buffer
RT-PCR: Reverse Transcription Polymerase Chain Reaction
JWXYS: Jia Wei Xiao Yao San
SJZT: Si Jun Zi Tang
SLBZS: Shen Ling Bai Zhu San
HXZQS: Huo Xiang Zheng Qi San
BW: Bai-Wei (*Radix Cynanchi Atrati*)

## References

[1] Bailly C Atractylenolides, essential components of Atractylodes-based traditional herbal medicines: Antioxidant, anti-inflammatory and anticancer properties Eur J Pharmacol 2021 891 173735 10.1016/j.ejphar.2020.173735 33220271

[2] Hoang le S Tran MH Lee JS Ngo QM Woo MH Min BS Inflammatory inhibitory activity of sesquiterpenoids from Atractylodes macrocephala rhizomes Chem Pharm Bull (Tokyo) 2016 64 507 11 10.1248/cpb.c15-00805 27150484

[3] Zhu B Zhang QL Hua JW Cheng WL Qin LP The traditional uses, phytochemistry, and pharmacology of Atractylodes macrocephala Koidz.: a review J Ethnopharmacol 2018 226 143 67 10.1016/j.jep.2018.08.023 30130541

[4] Huang KC Yen HR Chiang JH Su YC Sun MF Chang HH Chinese herbal medicine as an adjunctive therapy ameliorated the incidence of chronic hepatitis in patients with breast cancer: a nationwide population-based cohort study Evid Based Complement Alternat Med 2017 2017 1052976 10.1155/2017/1052976 29234362 PMC5682887

[5] Li S Chen X Shi H Yi M Xiong B Li T Tailoring traditional Chinese medicine in cancer therapy Mol Cancer 2025 24 27 39838407 10.1186/s12943-024-02213-6 PMC11749133

[6] Mayo B Penroz S Torres K Simón L Curcumin administration routes in breast cancer treatment Int J Mol Sci 2024 25 11492 10.3390/ijms252111492 39519045 PMC11546575

[7] Barcelos KA Mendonça CR Noll M Botelho AF Francischini CRD Silva MAM Antitumor properties of curcumin in breast cancer based on preclinical studies: a systematic review Cancers (Basel) 2022 14 2165 10.3390/cancers14092165 35565294 PMC9099919

[8] Bayet-Robert M Kwiatkowski F Leheurteur M Gachon F Planchat E Abrial C Phase I dose escalation trial of docetaxel plus curcumin in patients with advanced and metastatic breast cancer Cancer Biol Ther 2010 9 8 14 10.4161/cbt.9.1.10392 19901561

[9] Kesarwani K Gupta R Mukerjee A Bioavailability enhancers of herbal origin: an overview Asian Pac J Trop Biomed 2013 3 253 66 10.1016/S2221-1691(13)60060-X 23620848 PMC3634921

[10] Othman AK El Kurdi R Badran A Mesmar J Baydoun E Patra D Liposome-based nanocapsules for the controlled release of dietary curcumin: PDDA and silica nanoparticlecoated DMPC liposomes enhance the fluorescence efficiency and anticancer activity of curcumin RSC Adv 2022 12 11282 92 10.1039/d2ra00071g 35425076 PMC8996248

[11] Zhang Y Xu L Li A Han X The roles of ZEB1 in tumorigenic progression and epigenetic modifications Biomed Pharmacother 2019 110 400 8 10.1016/j.biopha.2018.11.112 30530042

[12] Baghban R Roshangar L Jahanban-Esfahlan R Seidi K Ebrahimi-Kalan A Jaymand M Tumor microenvironment complexity and therapeutic implications at a glance Cell Commun Signal 2020 18 59 10.1186/s12964-020-0530-4 32264958 PMC7140346

[13] Jia X Yan B Tian X Liu Q Jin J Shi J CD47/SIRPα pathway mediates cancer immune escape and immunotherapy Int J Biol Sci 2021 17 3281 7 10.7150/ijbs.60782 34512146 PMC8416724

[14] Song YC Lee DY Yeh PY A novel Chinese herbal and corresponding chemical formula for cancer treatment by targeting tumor maintenance, progression Metastasis Front Pharmacol 2022 13 907826 10.3389/fphar.2022.907826 35721174 PMC9204638

[15] Long F Wang T Jia P Wang H Qing Y Xiong T Anti-tumor effects of Atractylenolide-I on human ovarian cancer cells Med Sci Monit 2017 23 9 10.12659/msm.902886 PMC5297331 28141785

[16] Liu H Zhu Y Zhang T Zhao Z Zhao Y Cheng P Anti-tumor effects of atractylenolide I isolated from Atractylodes macrocephala in human lung carcinoma cell lines Molecules 2013 18 13357 68 10.3390/molecules181113357 24172243 PMC6270531

[17] Chen JL Chang CJ Wang JY Wen CS Tseng LM Chang WC In vitro and in vivo effects of Jia-Wei-Xiao-Yao-San in human breast cancer MCF-7 cells treated with tamoxifen Integr Cancer Ther 2014 13 226 39 10.1177/1534735414520970 24525674

[18] Tan W Lu J Huang M Li Y Chen M Wu G Anticancer natural products isolated from Chinese medicinal herbs Chin Med 2011 6 27 10.1186/1749-8546-6-27 21777476 PMC3149025

[19] Zhang Y Liang Y He C Anticancer activities and mechanisms of heat-clearing and detoxicating traditional Chinese herbal medicine Chin Med 2017 12 20 10.1186/s13020-017-0140-2 28702078 PMC5506596

[20] Xiang Y Guo Z Zhu P Chen J Huang Y Traditional Chinese medicine as a cancer treatment: modern perspectives of ancient but advanced science Cancer Med 2019 8 1958 75 10.1002/cam4.2108 30945475 PMC6536969

[21] Grantab RH Tannock IF Penetration of anticancer drugs through tumour tissue as a function of cellular packing density and interstitial fluid pressure and its modification by bortezomib BMC Cancer 2012 12 214 10.18632/oncotarget.26267 22672469 PMC3407510

[22] Libutti SK Tamarkin L Nilubol N Targeting the invincible barrier for drug delivery in solid cancers: interstitial fluid pressure Oncotarget 2018 9 35723 5 10.18632/oncotarget.2626 30515264 PMC6254664

[23] Primeau AJ Rendon A Hedley D Lilge L Tannock IF The distribution of the anticancer drug Doxorubicin in relation to blood vessels in solid tumors Clin Cancer Res 2005 11 8782 8 10.1158/1078-0432.CCR-05-1664 16361566

[24] Trédan O Galmarini CM Patel K Tannock IF Drug resistance and the solid tumor microenvironment J Natl Cancer Inst 2007 99 1441 54 10.1093/jnci/djm135 17895480

[25] Wang Y Liu B Fu X Tong T Yu Z Efficacy and safety of Si-Jun-Zi-Tang-based therapies for functional (non-ulcer) dyspepsia: a meta-analysis of randomized controlled trials BMC Complement Med Ther 2021 21 11 10.1186/s12906-020-03176-z 33407405 PMC7788807

[26] Liu L Ge F Yang H Shi H Lu W Sun Z Xiao-Yao-San Formula improves cognitive ability by protecting the hippocampal neurons in ovariectomized rats Evid Based Complement Alternat Med 2020 2020 4156145 10.1155/2020/4156145 32655660 PMC7321526

[27] Zhang Y Han M Liu Z Wang J He Q Liu J Chinese herbal formula xiao yao san for treatment of depression: a systematic review of randomized controlled trials Evid Based Complement Alternat Med 2012 2012 931636 10.1155/2012/931636 21869903 PMC3159992

[28] Chen HY Huang BS Lin YH Su IH Yang SH Chen JL Identifying Chinese herbal medicine for premenstrual syndrome: implications from a nationwide database BMC Complement Altern Med 2014 14 206 10.1186/1472-6882-14-206 24969368 PMC4099402

