# Supplementary material for: Evaluation of Bai-Zhu (Atractylodes macrocephala)-based herbal formulae in breast cancer: Implications for metastasis via Zeb1 and Slug modulation
Source: Biomedicine (Taipei). 2026 Jun 1;16(2):24–34. doi: 10.37796/2211-8039.1704 (PMC13387403; doi:10.37796/2211-8039.1704)
Supplement: Supplementary file 1 [file bmed-16-02-024-s001.doc]

**The composition of herbal formula.**

This information is from Sun Ten Pharmaceutical Co. (Taiwan).

1. Si Jun Zi Tang: 12 g of material is made from the extract of 6 g Ginseng Radix, 6 g Poria cocos, 6 g Atractylodes macrocephala Koidz., 3 g Radix Glycyrrhizae preparata, 3 g Jujubae Fructus, and 2 g Zingiberis Rhizoma Recens.
2. Jia Wei Xiao Yao San: 15 g of material is made from the extract of 4 g Radix Angelicae sinensis, 4 g Atractylodes macrocephala Koidz., 4 g Poria cocos, 2 g Radix Glycyrrhizae preparata, 4 g Bupleuri Radix, 4 g Radix Paeoniae Alba, 2.5 g Cortex Moutan, 2.5 g Gardeniae Fructus, 2 g Menthae Herba, and 4 g Zingiberis Rhizoma preparata.
3. Shen Ling Bai Zhu San: 10.5 g of material is made from the extract of 2.3 g Lablab Semen Album, 3 g Ginseng Radix, 3 g Poria cocos, 3 g Rhizome Atractylodis macrocaphalae, 3 g Radix Glycyrrhizae preparata, 3 g Dioscoreae Rhizoma, 1.5 g Nelumbinis Semen, 1.5 g Platycodonis Radix, 1.5 g Coicis Semen, 1.5 g Amomi Fructus, and 1.5 g Jujubae Fructus.
4. Huo Xiang Zheng Qi San: 15 g of material is made from the extract of 3 g Arecae Pericarpium, 2.25 g Poria cocos, 2.25 g Angelicae Dahuricae Radix, 2.25 g Perillae Folium, 1.5 g Citri Reticulatae Pericarpium, 1.5 g Platycodonis Radix, 1.5 g Atractylodes macrocephala Koidz., 1.5 g Cortex Magnoliae officinalis, 2 g Rhizoma Pinelliae fermertatae, 1 g Radix Glycyrrhizae preparata, 3 g Pogostemonis Herba, 3 g Zingiberis Rhizoma Recens, and 1 g Jujubae Fructus.

**Preparation of the formulae**

Si Jun Zi Tang and Jia Wei Xiao Yao San combined each herb with a relative ratio of each herb as follows:

Si Jun Zi Tang: Ginseng Radix: Poria cocos: Atractylodes macrocephala Koidz.: Radix Glycyrrhizae preparata at a ratio of 2:2:2:1.

Jia Wei Xiao Yao San: Radix Angelicae sinensis: Atractylodes macrocephala Koidz.: Poria cocos: Bupleuri Radix: Radix Paeoniae Alba: Radix Glycyrrhizae preparata: Cortex Moutan: Gardeniae Fructus: Menthae Herba: Zingiberis Rhizoma preparata as 2:2:2:2:2:1:1.25:1.25:1:2.

The indicated concentration of the formula was the amount of Atractylodes macrocephala Koidz. at 10 mg/ml in pure water, and the other formulae were included according to the relative ratio to Atractylodes macrocephala Koidz.. All of the herbs that were prepared in pure water were autoclaved, and the solution was stored at -20 °C.
