# Supplementary material for: Evaluation of Bai-Zhu (Atractylodes macrocephala)-based herbal formulae in breast cancer: Implications for metastasis via Zeb1 and Slug modulation
Source: Biomedicine (Taipei). 2026 Jun 1;16(2):24–34. doi: 10.37796/2211-8039.1704 (PMC13387403; doi:10.37796/2211-8039.1704)
Supplement: Supplementary file 2 [file bmed-16-02-024-s002.pdf]

Supplementary Figure S1

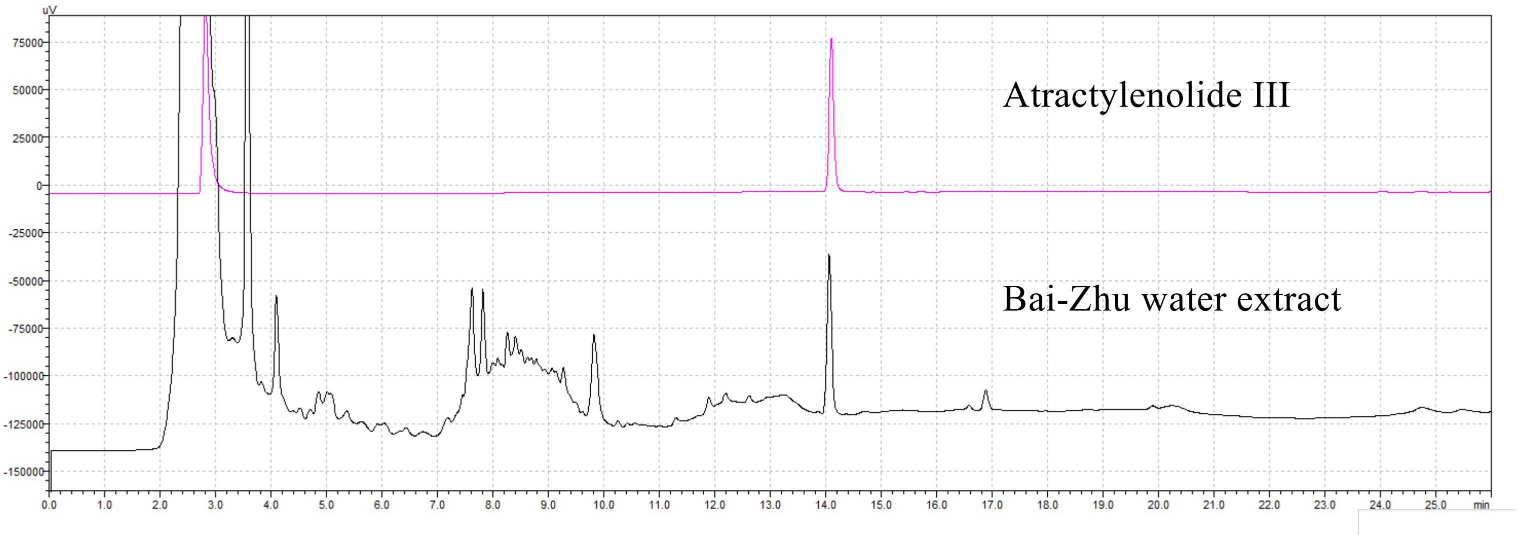

Supplementary Fig. S1. The HPLC profiles of the chemical components of the Bai-Zhu water extract (black) and the standard atractylenolide III (pink) were recorded, with UV detection set at a wavelength of 220 nm. A SHIMADZU system consisting of a UV-Vis detector (SPD-40), a binary pump (LC-40D), a degasser (DGU-403), and a column oven (CTO-40S), along with an HPLC column (4.6 × 250 mm I.D. with a 5 μm particle size, COSMOSIL® 5C18–MS–II) was used for chemical HPLC profiling. The mobile phases contained two eluents, including acetonitrile (MeCN) and an aqueous solution with the following solvent program: 20% MeCN for 3 min, 20–50% MeCN for 3 min, 50–80% MeCN for 9 min, 85% MeCN for 5 min, 85–100% MeCN for 10 min, and 100% MeCN for 1 min. A total of 10 μL and 30 μL of injected sample volumes and concentrations of 1.4 mg/mL atractylenolide III and 439.5 mg/mL Bai-Zhu water extract were used, respectively. The column oven was set at 30 °C, and the absorbance of the UV spectrum was read at 220 nm. In this study, the flow rate of the mobile phase was 1.0 mL/min.

Supplementary Figure S2

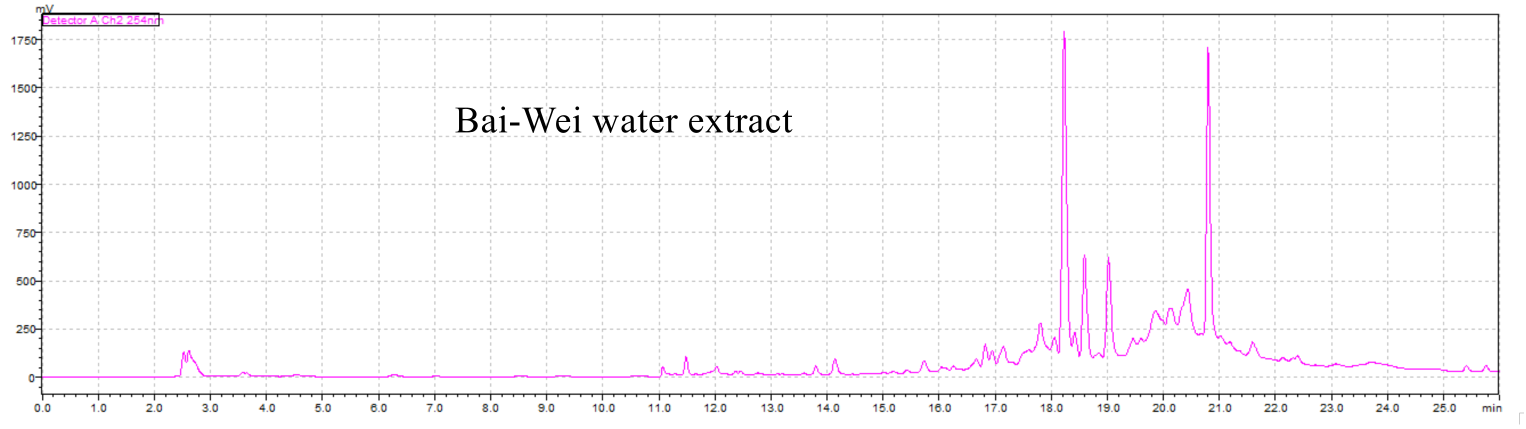

Supplementary Fig. S2. HPLC profile of the chemical components of the Bai-Wei water extract. UV detection was performed at a wavelength of 254 nm. A SHIMADZU system consisting of a UV-Vis detector (SPD-40), a binary pump (LC-40D), a degasser (DGU-403), and a column oven (CTO-40S), along with an HPLC column (4.6 × 250 mm I.D. with a 5 µm particle size, COSMOSIL® 5C18–MS–II) was used for chemical HPLC profiling. The mobile phases contained two eluents, including acetonitrile (MeCN) and an aqueous solution with the following solvent program: 0% MeCN for 5 min, 0–20% MeCN for 10 min, 20–100% MeCN for 15 min, and 100% MeCN for 1 min. A 30 µL injected sample volume and 186.5 mg/mL Bai-Wei water extract concentrations were used. The column oven was set at 30 °C, and the absorbance of the UV spectrum was read at 254 nm. In this study, the flow rate of the mobile phase was 1.0 mL/min.
